# Methods to improve the accuracy of next-generation sequencing

**DOI:** 10.3389/fbioe.2023.982111

**Published:** 2023-01-20

**Authors:** Chu Cheng, Zhongjie Fei, Pengfeng Xiao

**Affiliations:** State Key Laboratory of Bioelectronics, School of Biological Science and Medical Engineering, Southeast University, Nanjing, China

**Keywords:** improvement, high accuracy, clinical application, future development, next-generation sequencing

## Abstract

Next-generation sequencing (NGS) is present in all fields of life science, which has greatly promoted the development of basic research while being gradually applied in clinical diagnosis. However, the cost and throughput advantages of next-generation sequencing are offset by large tradeoffs with respect to read length and accuracy. Specifically, its high error rate makes it extremely difficult to detect SNPs or low-abundance mutations, limiting its clinical applications, such as pharmacogenomics studies primarily based on SNP and early clinical diagnosis primarily based on low abundance mutations. Currently, Sanger sequencing is still considered to be the gold standard due to its high accuracy, so the results of next-generation sequencing require verification by Sanger sequencing in clinical practice. In order to maintain high quality next-generation sequencing data, a variety of improvements at the levels of template preparation, sequencing strategy and data processing have been developed. This study summarized the general procedures of next-generation sequencing platforms, highlighting the improvements involved in eliminating errors at each step. Furthermore, the challenges and future development of next-generation sequencing in clinical application was discussed.

## 1 Introduction

DNA sequencing has become a conventional technique in modern biological research ever since Sanger established the “dideoxy chain termination sequencing method” in 1977 (Sanger et al., 1977). The launch of the next-generation sequencing (NGS) platforms has greatly reduced the cost of DNA sequencing, and has had a huge impact on research in contemporary biology, medicine and other fields (Metzker, 2005; Shendure and Ji, 2008; Metzker, 2009; Scholz et al., 2012; Schrijver et al., 2012). NGS, which involves massively parallel sequencing of multiple templates in a single sequencing run, generates large amounts of data (Mardis, 2011). NGS is now the current mainstream sequencing platform employed for sequencing as a clinical tool and one of the major sources of medical big data (Drmanac, 2011; Goodwin et al., 2016).

Although theoretically, any mutations should be detectable when sequencing depth is large enough, the practical limits of detection are caused by errors introduced during sample preparation and sequencing (Gundry and Vijg, 2012). In addition to base misincorporations and allelic frequencies skewing that can result from PCR amplification, the additional errors that arise during cluster amplification, cycle sequencing, and image analysis, ∼1% of bases are incorrectly detected, depending on the specific platform. The high error rate of NGS remains a major obstacle to its large-scale application (Lin et al., 2012). For example, due to high error rate of NGS technologies, high-coverage assembly is required to eliminate errors, resulting in low-abundance mutations being lost as sequencing errors. In addition, methylation haplotype analysis, which can not only detect cancer but also locate the location of tumor growth and onset, also depends on the accuracy of a single read (Metzker, 2010). Roche/454 displays an error rate of 1% (Rieber et al., 2013); Illumina sequencing runs consistently display a base-pair error rate of 0.26%–0.8% (Van Dijk et al., 2014); Ion Torrent displays an error rate of 1.78% (Mascher et al., 2013); the dual-base encoding used by SOLiD is able to lower this error rate to about 0.06% (Ronchi et al., 2012); PacBio and Nanopore are not discussed in this paper. These error rates are still higher than that of Sanger sequencing (0.001%) (Hoff, 2009; Xin et al., 2012). Although the error rate seems low at one per hundred or one per thousand bases, given the size of human genome, this could lead to accumulated errors which are not negligible and creates great obstacle for mutation detection. Some false variants are very similar to real somatic mutations and rare mutations, and downstream validation of these false positive variants can be costly, so it is very important to improve the accuracy of sequencing.

A variety of improvements at the level of template preparation, sequencing strategy and data processing have been developed in order to improve sequencing accuracy. In this review, an overview of the procedure for NGS is first outlined, and the improvement involved in each step for error elimination is then discussion. Finally, challenges and future research trends of NGS in clinical applications are also discussed.

## 2 Overview of next-generation sequencing (NGS)

**FIGURE 1:**
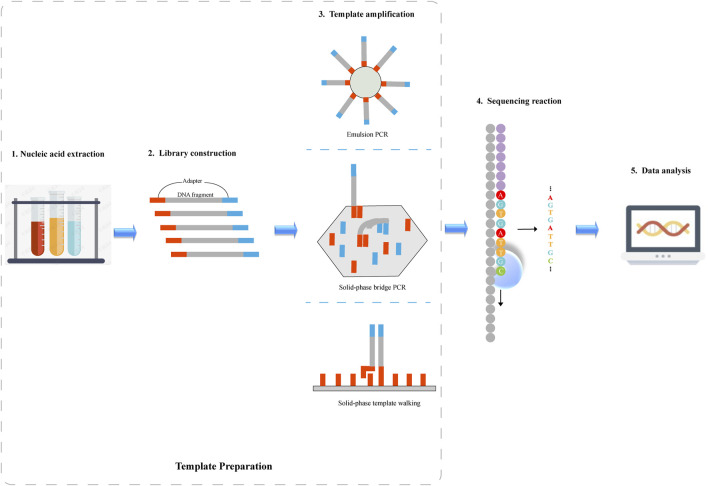
Schematic diagram of next-generation sequencing. 1) Nucleic acid is extracted from the sample. 2) DNA fragments are sheared into smaller fragments through sonication or enzymatic fragmentation, and then the sheared DNA is ligated to adapter sequences where DNA sequencing is initiated. 3) Following library preparation, DNA fragments are amplified by PCR. Depending on the sequencing platform, emulsion PCR, solid-phase bridge PCR or solid-phase template walking is used to generate clonal template populations. 4)DNA sequence information is obtained by a sequencing platform. 5) The sequencing data is analyzed by various algorithms.

There are five major steps in regard to NGS system operation: nucleic acid extraction, library construction, template amplification, sequencing reaction and data analysis, as illustrated in Figure 1. Nucleic acid extraction, library construction, and template amplification belong to template preparation.

### 2.1 Nucleic acid extraction

Nucleic acid is extracted from the sample. The protocol is not universal but depends upon the sample source and type of study to be conducted (Metzker, 2009; Fierer et al., 2012). Especially for environmental samples, further pretreatments are required to remove impurities (Pawlowski et al., 2022).

### 2.2 Library construction

DNA Library preparation begins by shearing isolated DNA fragments into smaller, random, overlapping fragments, and then the sheared DNA is ligated to adapter sequences where DNA sequencing is initiated. The isolated DNA is fragmented in the range from 150 to 800 bp, depending on the platform used, which may be achieved mechanically (by passing the DNA through a narrow passage), through sonication or enzymatic fragmentation (Caruccio, 2011; Knierim et al., 2011; Parkinson et al., 2012). RNA library is prepared by capturing mRNA, randomly fragmenting and synthesizing complementary DNA (cDNA), which is then followed by ligating to adapters for clonal amplification and sequencing (Frias-Lopez et al., 2008; Shi et al., 2011; Lesniewski et al., 2012).

### 2.3 Template amplification

Following library preparation, DNA fragments are amplified on a solid phase (either a glass slide or a microbead) by a polymerase-mediated reaction. Depending on the sequencing platform, emulsion PCR (emPCR), solid-phase bridge PCR or solid-phase template walking is used to generate clonal template populations (Dressman et al., 2003; Fedurco et al., 2006). In emPCR, DNA molecules are immobilized on magnetic beads in order to ensure that only one DNA molecule is contained on a magnetic bead. Each magnetic bead is independently amplified, and magnetic beads do not contaminate each other (Shendure et al., 2005). The solid-phase bridge and template walking PCR methods covalently bind a high concentration of primers to a suitable solid surface, forming clusters after amplification (Fedurco et al., 2006). Solid-phase amplification can generate 100 to 200 million separate clusters and ensure that the DNA molecules contain free ends so that the DNA molecules are able to bind to the universal sequencing primers and enter sequencing.

### 2.4 Sequencing reaction

Following amplification, DNA sequence information is obtained using a sequencing platform. Current commercial NGS platforms fall into two broad categories: based on sequencing-by-synthesis (SBS; e.g., Roche/454, Illumina/Solexa, Ion Torrent) and based on sequencing-by-ligation (SBL; e.g., SOLiD). SBL-based method performs DNA sequencing using a probe sequence attached to a fluorophore that hybridizes to the target DNA and is ligated to an adjacent oligonucleotide for imaging. The fluorescent signal indicates the identity of bases complementary to specific locations within the probe. SBS-based method uses polymerase to extend a new DNA strand and identifies the incorporated oligonucleotides during the strand synthesis. Different NGS platforms produce different kinds of errors.

Roche/454 is the first commercially successful SGS system (Margulies et al., 2005). This sequencer has a relatively fast sequencing speed and long read length, though it lacks single-base accuracy in measuring homopolymers larger than 6–8 bp (Loman et al., 2012; Forgetta et al., 2013). Additionally, its cost in comparison to other NGS platforms is high. Illumina/Solexa platform accounts for the largest market share of sequencing instruments compared to other platforms. It can fully address the issue in homopolymer sequencing, though a tendency towards substitution errors in the AT-rich regions and CG-rich regions exists (Dohm et al., 2008; Harismendy et al., 2009; Minoche et al., 2011; Nakamura et al., 2011). Ion Torrent is a NGS platform that utilizes semiconductors. Similar to Roche/454 system, the pH change detected by the sensor has poor linearity with respect to the number of nucleotides incorporated in a single reaction cycle, thus limiting its accuracy in measuring the homopolymer regions (Pu and Xiao, 2017). SOLiD is a NGS sequencer that is based on SBL, of which a single sequencing cycle is composed of various two-base encoded probes that bind, ligate, image and cleave. It has the highest accuracy among NGS platforms, but its short read length increases the difficulty of genome assembly.

### 2.5 Data analysis

NGS sequencers can generate large volumes of data in a single experiment. Accordingly, a series of complex algorithms need to be developed continuously for sequence assemble, variant calling, and data visualization. NGS data analysis comprises three basic stages: 1) the conversion of sequencing chemistry to base information, providing base detection and associated mass scores that reflect the primary structure of DNA or RNA strand; 2) the alignment and assembly of DNA or RNA fragments, providing a complete sequence for the sample so that genetic variants can be identified; 3) the interpretation of genetic variations to gain knowledge and insights into basic biology.

## 3 Improvements in template preparation

In regard to NGS platforms, the template needs to be amplified prior to conducting sequencing. However, the use of PCR adds the potential for several serious artifacts (Hughes and Totten, 2003; Kanagawa, 2003): First, new base errors are introduced by polymerase during PCR amplification (Meyerhans et al., 1990). Errors that occur in the previous rounds of PCR amplification can be amplified in subsequent PCR processes, which may bring about false mutations; second, when the premature termination products start the next round of synthesis, artificial recombination occurs during amplification, which may obscure the connection between the two sequences polymorphism (Yang et al., 1996); third, for real mutations, the PCR reaction may amplify more aggressively against DNA templates containing one base (PCR bias). Therefore, if the reaction system is strongly biased to the amplification of the template containing reference allele, the information of the mutant base will become smaller, leading to a false negative (Liu et al., 1996; Goren et al., 2010). Overall, PCR has a negative effect on both the detection of real variation and the determination of individual genotypes.

In order to overcome errors that may presumably result from mutations introduced by PCR during template preparation on the instrument itself, tagging techniques that create a unique tag for the DNA template before amplification can be used (Cervantes et al., 2002; Miner et al., 2004; McCloskey et al., 2007; Parameswaran et al., 2007; Hiatt et al., 2010; Fu et al., 2011; Jabara et al., 2011; Kinde et al., 2011; Kivioja et al., 2012; Schmitta et al., 2012). Tags for DNA sequencing have guaranteed error correction capability as all amplicons obtained from a particular starting DNA molecule can be clearly identified (Krishnan et al., 2011). Any change in the sequence or copy number of identically tagged sequencing can be considered as a technical error. Tags, also known as barcodes or indexes, can be assigned to nucleic acid fragments using a variety of methods, which include the use of unique random shear points as template tags (Hiatt et al., 2010), the introduction of exogenous tags into the template by PCR (McCloskey et al., 2007; Parameswaran et al., 2007; Jabara et al., 2011; Kinde et al., 2011), and the introduction of exogenous tags into the template by ligation (Cervantes et al., 2002; Miner et al., 2004; Fu et al., 2011; Kivioja et al., 2012; Schmitta et al., 2012).

**FIGURE 2:**
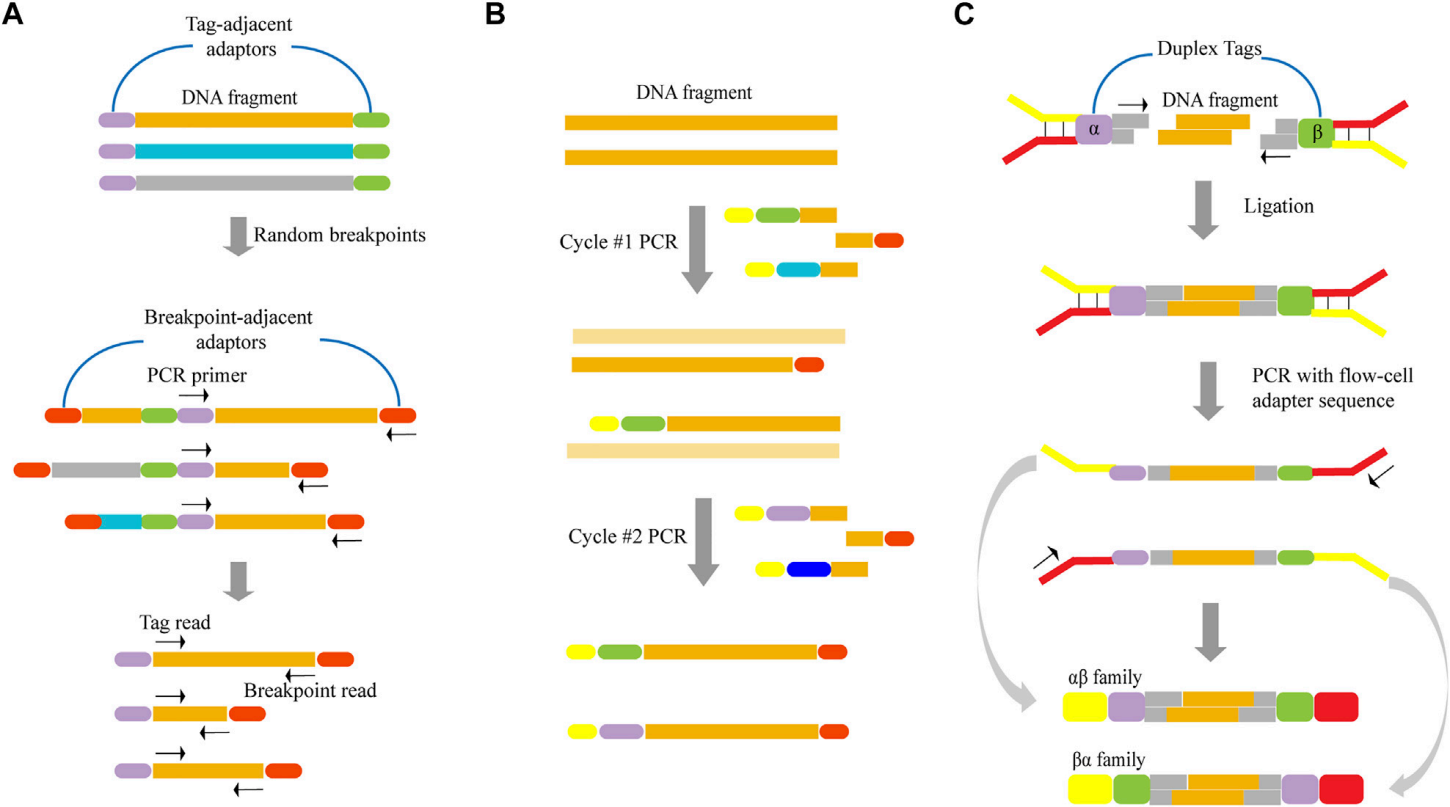
Schematics of various template tagging methods. **(A)** Using unique random shear points as template tags. Lone DNA fragments are ligated to tag-adjacent adaptors, diluted and PCR-amplified. PCR products are sheared through sonication and ligated to breakpoint-adjacent adaptors. Next, a second round of PCR amplification is conducted, where one end corresponds to a tag-adjacent adaptor and the other corresponds to a breakpoint-adjacent adaptor. **(B)** Introducing exogenous tags into the template by PCR. The tagged template requires two rounds of PCR in which one primer has a random DNA sequence that forms the unique tag (various colored bars). Both primers have sequences that permit universal amplification in the next step (yellow and red bars). **(C)** Introducing exogenous tags into the template by ligation. The DNA fragment is ligated to adapters that contain duplex tags (purple and green bars), which becomes labeled with two distinct tags. Following PCR amplification, two kinds of tagged families (αβ family and βα family) are produced.

In terms of unique random shear points as template tags, one of the two reads from a paired-end read serves as a sequence tag to identify short read groups of shared clone origin, that is, deriving from the same DNA fragment (Figure 2A). First, DNA is sheared to a relatively long length, after which long DNA fragments are ligated to adaptors. Following dilution and amplification of these fragments, PCR products are sheared through sonication and ligated to breakpoint-adjacent adaptors. Next, a second round of PCR amplification is conducted, where one end corresponds to a tag-adjacent adaptor and the other corresponds to a breakpoint-adjacent adaptor. The resulting amplicons contain a population of nested sub-libraries derived from the original long-range library. Tag-adjacent adapters provide access to genomic sequences corresponding to the ends of long fragments. Since this end sequence will be identical to the amplicon derived from the same long fragment, it can serve as a tag in identifying molecular clones. After pair-end sequencing, the reads initiated by the tag-adjacent adapters identify the original long DNA fragment, while the reads initiated by the breakpoint-adjacent adapters represent the sequence of the cleavage-determined breakpoint in the fragment. Hiatt et al. (2010) reported a reduction in the error rate of ∼10-fold by employing this tagging method, in which the longest error-free sequence was showed to be up to 680 bp. This approach achieved a low overall error rate of one per 400 bp.

When exogenous tags are used, they can be introduced by PCR and ligation. This method of ligation directly attaches the tags to the template by ligase. When introducing exogenous tags by PCR (Figure 2B), two rounds of PCR are required. First, DNA is amplified with a set of gene-specific primers. One primer has a random DNA sequence that forms the unique tag, and both primers have sequences that permit universal amplification in the next step. Two different tag assignment cycles produce two fragments—each with a different tag—from each double-stranded template molecule, as shown in Figure 2B. Kinde et al. (2011) demonstrated that this approach, which is based on labeling single-strand DNA fragments with exogenous tags, could reduce the error rate by about 20-fold, and allow for an observed mutation frequency of normal human genomic DNA of ∼0.001% mutations/bp.

However, almost all of the reported tagging methods were tagged for single-stranded DNA. Since the base change is propagated to all subsequent PCR copies if an artificial mutation is introduced in the first round of PCR, this may result in errors that are undetectable even with techniques that label single-stranded DNA. In order to overcome this limitation, Schmitta et al. (2012) discussed a technique that involved simultaneously labeling double-strand DNA. Here, the DNA fragment was ligated to adapters that contain duplex tags, which were then labeled with two distinct tags (Figure 2C). Following PCR amplification, two kinds of tagged families (αβ family and βα family) were produced from each DNA fragment. As the two strands were complementary, true mutations that presented on both strands of the DNA fragment appeared in all members of the family pair. In contrast, PCR or sequencing errors resulted in mutations in only one strand and can be discounted as technical error. As a result, they determined that this method could result in a ∼20-fold improvement in accuracy relative to standard Illumina sequencing. Furthermore, it achieved a theoretical background error rate of less than one artifactual error per 10^9^ nucleotides.

Using high-fidelity enzymes for amplification is another way to reduce errors during template preparation. Multiple displacement amplification (MDA), another isothermal amplification technique, replaces Bst polymerase with phi29 polymerase of high fidelity to reduce misincorporations during amplification (Chen et al., 2014). MDA is more commonly used in whole genome amplification, through it is challenged by uneven amplification (Dean et al., 2001; Lasken, 2009; Hou et al., 2015). Many researchers have proposed improvement methods, including the optimization of reagents and conditions as well as the use of microfluidic devices to physically separate the entire reaction system into many tiny chambers or droplets. Among them, Li et al. (2017) proposed a novel MDA method, called μcMDA, which decentralizes MDA reagents throughout a one-dimensional slender tube. They demonstrated that this method can significantly improve the uniformity of amplification, enabling the accurate detection of single nucleotide variation with higher efficiency and sensitivity.

Another improvement for template preparation is amplification-free sequencing. Kozarewa et al. (2009) proposed an amplification-free method pertaining to Illumina sequencing-library preparation. Here, unlike the standard Illumina adapters, PCR-free adapters contained additional sequences, allowing templates to directly hybridize to flow cell surfaces. Fragments that were incompletely attached were shown to be inert in the cluster amplification step. Therefore, it was not necessary to retain the PCR step so as to enrich the correct ligated fragment. However, in order to obtain optimal cluster density, it was necessary to precisely quantify only the template fragments with an adapter at either end. The authors of this study illustrated that this method can reduce the incidence of duplicate sequences and finally improve read mapping and SNP calling while helping *de novo* assembly. Due to the impact of amplification on the sequencing results, amplification-free sequencing, including NGS-based amplification-free sequencing and single-molecule sequencing, may be more likely to be adopted in the future.

## 4 Improvements in sequencing strategy

Comparison between NGS and Sanger sequencing has demonstrated that NGS is superior in terms of throughput and sequencing efficiency. As far as error rate and read length is concerned, however, Sanger sequencing remains the gold standard (Table 1). Among NGS platforms, the accuracy of the original base data obtained by SOLiD platform is greater than 99.94%, though accuracy can reach 99.999% with the sequencing depth of 15×, which is the highest accuracy in NGS platforms (Ronchi et al., 2012).

**TABLE 1:** Comparison of sequencing indexes and characteristics of NGS platforms.

Sequencing platforms	Accuracy (%)	Read length	Maximum output/run	Application
454 GS FLX	99	700 bp	0.5 Gb	Small genomic DNA and RNA research
MiniSeq	99.2	2 bp × 150 bp	7.5 Gb	Low throughput sequencing of target DNA and RNA
MiSeq	99.2	2 bp × 300 bp	15 Gb	Amplicon, target DNA and RNA sequencing
NextSeq	99.2	2 × 150 bp	120 Gb	Exome, transcriptome sequencing or resequencing
HiSeq	99.74	2 bp × 150 bp	1,500 Gb	Large-scale genome, exome, and transcriptome sequencing
Hiseq X	99.74	2 bp × 150 bp	1800 Gb	Large-scale whole genome sequencing
Ion Torrent	98.22	200 bp	10 Gb	Small genomic DNA and RNA research
SOLiDv4	99.94	50 + 50 bp	120 Gb	whole genome resequencing, targeted resequencing, transcriptome research
Sanger 3730xl^a^	99.999	900 bp	84 Kb	Look for specific genetic mutations associated with disease

^a^ Sanger sequencing is the first-generation sequencing.

**FIGURE 3:**
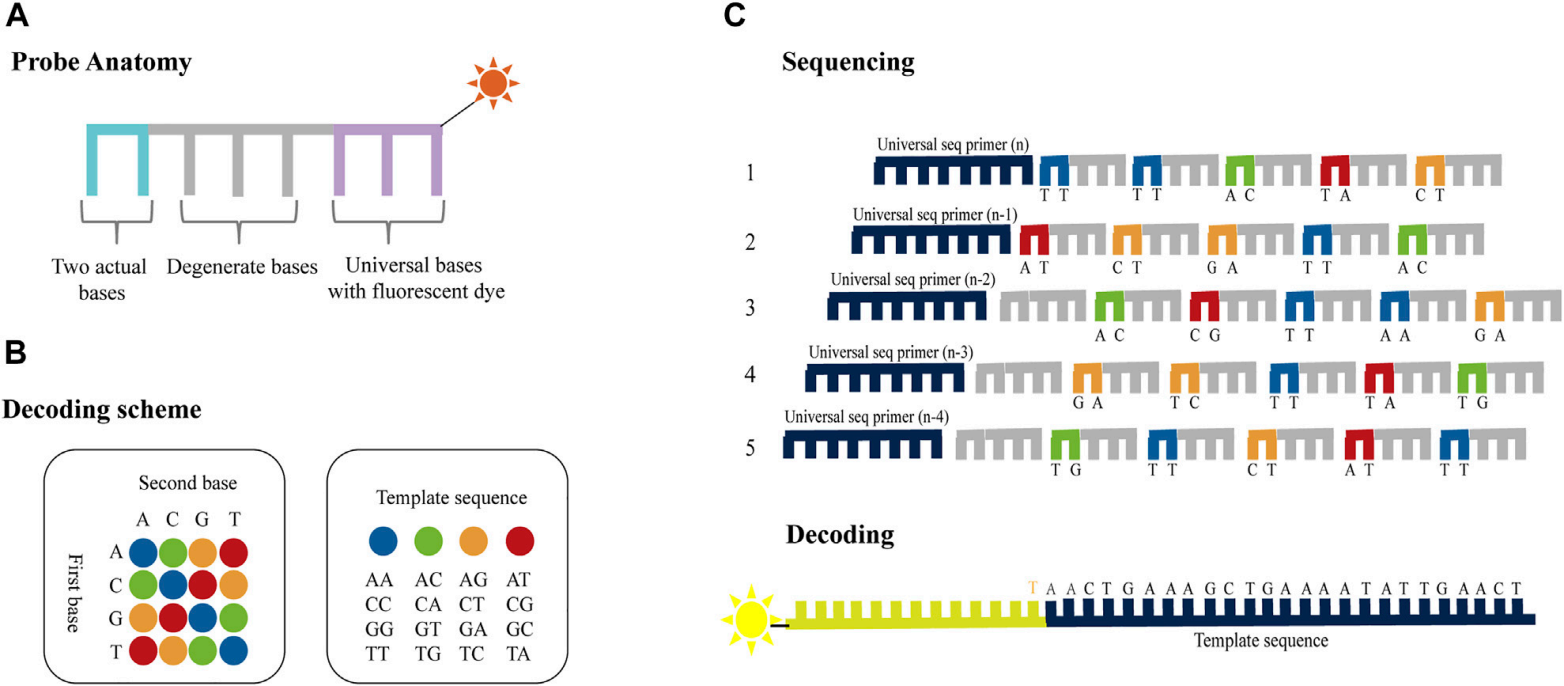
Schematics of dual-base sequencing based on SBL. **(A)** Two-base-encoded probe consists of two actual bases followed by three degenerate bases and three universal bases which are attached to a fluorescent label. **(B)** The decoding scheme of SOLiD platform. The four different colored probes represent 16 base pairs, respectively. **(C)** The probes are ligated with the primers using a DNA ligase and the fluorescent signal is detected to identify the first two bases in each fragment. Then the terminal degenerate bases and the fluorescent label are cleaved after detection thereby preparing the system for another round of ligation. The process is repeated until two out of every five bases are identified. The template sequence information can be deduced through five rounds of ligation reactions using ladder primers sets.

SOLiD platform utilizes two-base-encoded probes, in which each fluorescent signal represents a dual-base (Valouev et al., 2008). The probe is eight bases long and consists of two bases followed by three degenerate bases and three universal bases which are attached to a fluorescent label (denoted as 3′-XXNNNZ*ZZ-5′, * represents the cleavage site; Figure 3A). Since the 16 possible dual-base combinations cannot individually be associated with spectrally resolvable fluorophores, four fluorescent signals are used, each representing a subset of the four dual-base combinations (Figure 3B). Sequencing chemistry includes five rounds of sequencing reaction initiated by five primers, and each round of sequencing reaction includes multiple ligation reactions (Figure 3C). The first round of sequencing is initiated by primer n. The probes are ligated with the primers using a DNA ligase and the fluorescent signal, which represents the first and second bases, is detected by fluorescence imaging. The probes have cleavable linkages attached to fluorescent label which can be cleaved after detection thereby preparing the system for another round of ligation. Notably, each round of sequencing identifies two bases out of every five bases. That is, the first time is the first, second position, while the second time is the sixth, seventh position, and so on. After a round of probe extension, all probes and anchors are removed and a second round of sequencing begins with primer n−1. The difference between primers n−1 and n is that they differ by one base in the position of pairing with the linker. In primer n−1, the sequencing position is moved to the 5′end by one base, so that the 0th, first, fifth, sixth, and so on positions can be determined. After five rounds of sequencing, the original color encodings that represent the sequence information can be obtained, and the specific base of the 0th bit is known, so the specific sequence can be obtained by decoding the color encodings.

**FIGURE 4:**
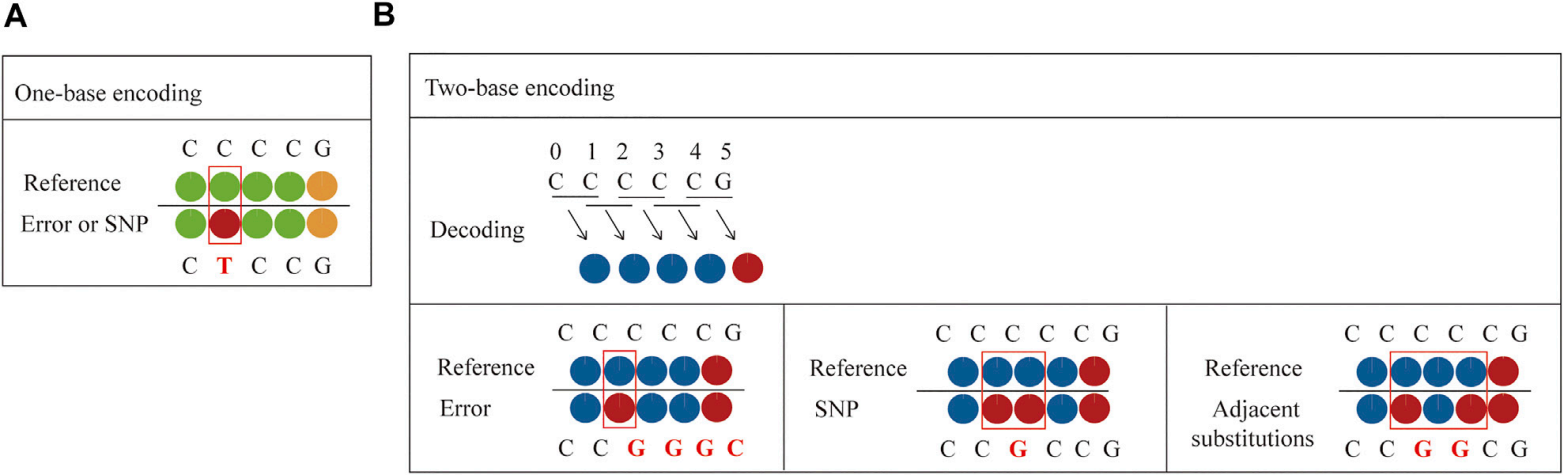
Comparison between one-base encoding and two-base encoding. **(A)** Characteristics of one-base encoding. Since one color corresponds to one base, when single-color change is observed, it may be a SNP or an error. **(B)** Characteristics of two-base encoding. When a single-color change is observed, all bases downstream from the color change will be different from the reference sequence. It indicates an error. When SNP is present in the sequence, it affects the color codes of two adjacent bases, resulting in two adjacent colors change. When adjacent substitutions are present in the sequence, the color code corresponding to the first substitution (C→G) and the previous base is different, and the color code corresponding to the second substitution (C→G) and the following base is also different, while the color code of these two substitutions can be the same or different.

Compared to one-base encoded sequencing (3′-XZ*XNNNZZ-5′), two-base encoding reduces the impact of connection efficiency and increases the sequencing read length, while reducing errors and distinguishing between errors and SNPs. This is because in one-base encoding, each color represents one base, so a single-color change can only indicate a SNP or an error, but it cannot make a specific judgment (Figure 4A). However, two-base encoding has the ability to distinguish between errors and SNPs. For example, when a single-color change is observed, all bases downstream from the color change will be different from the reference sequence. Therefore, single-color change indicates an error. Moreover, when SNP is present in the sequence, it will affect the color codes of two adjacent bases, resulting in two consecutive colors change. Thus, two colors change indicates a SNP. Meanwhile, when adjacent substitutions are present in the sequence, the color code corresponding to the first substitution (C→G) and the previous base is different, and the color code corresponding to the second substitution (C→G) and the following base is also different, while the color code of these two substitutions can be the same or different (Figure 4B). Thus, two or three colors change indicates two adjacent substitutions. Therefore, dual-base interrogation eases the discrimination between system errors and true SNPs by aligning color codes of reads against that of reference. Therefore, in the sequencing process, the use of dual-base sequencing strategy, where each sequencing run provides only an ambiguous sequence with partially defined base composition, can provide an inherent proofreading function, thereby reducing errors in the original data. Although SOLiD is limited in practical applications due to reaction time, read length, and so forth, high accuracy is the goal of NGS platforms.

**FIGURE 5:**
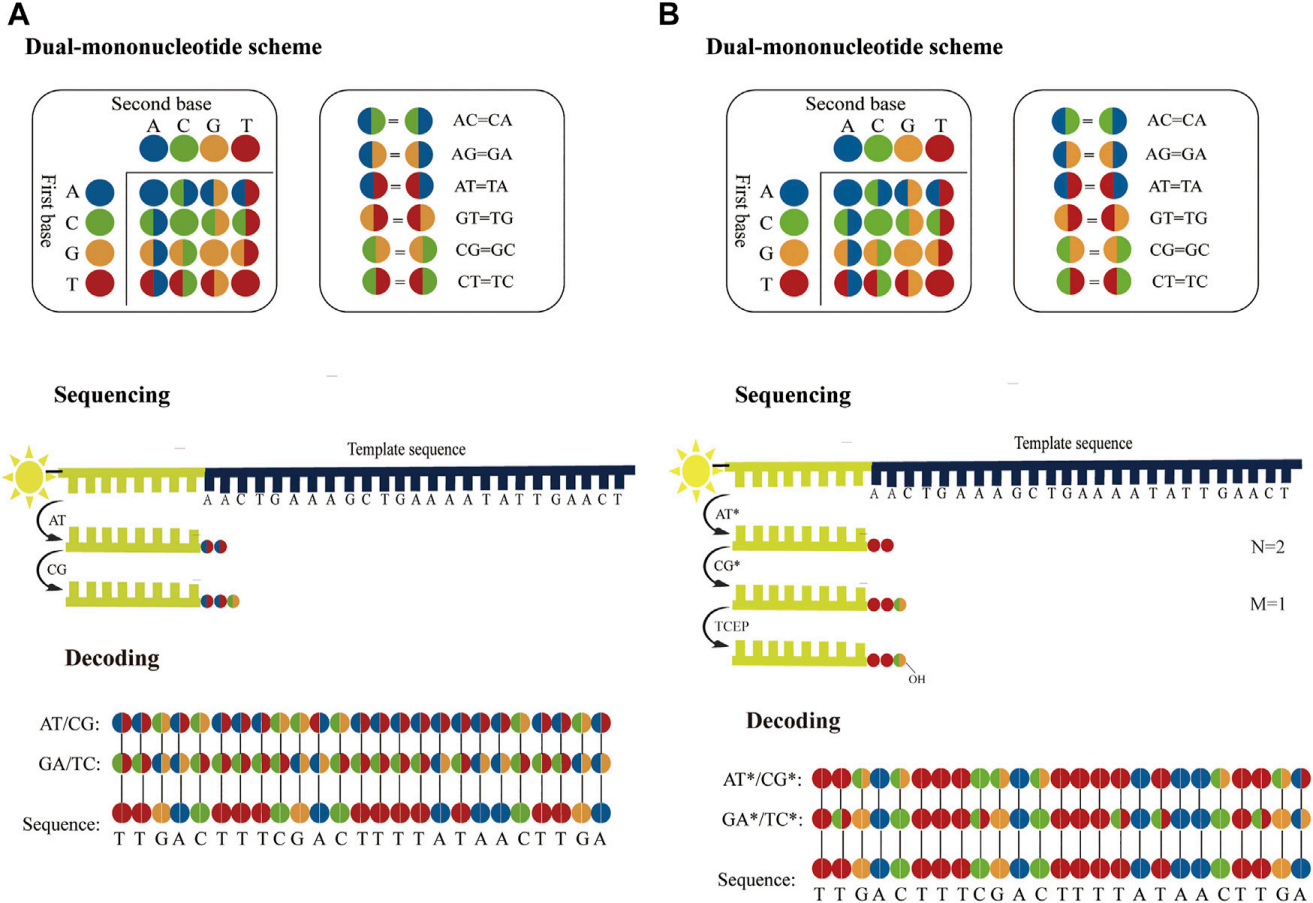
Schematics of dual-base sequencing based on SBS. **(A)** The schematic of real-time decoding sequencing technology. A mixture of two bases is added to a reaction cycle. The dual-base mixture allows any complementary bases to be incorporated to the synthetic strand, and releases an equivalent amount of ppi with base extended. When the template is interrogated by using AT/CG and GA/TC in two parallel sequencing runs, two sets of encodings can be obtained, from which the sequence can be deduced. **(B)** The schematic of correctable decoding sequencing technology. The template is interrogated by a mixture of two types of nucleotides, natural nucleotide and CRT. Each sequencing cycle consists of nucleotide extension, signal detection and deprotection. In the first extension reaction, a mixture of AT* is added and the number of incorporated bases “N = 2” is obtained. The unbonded nucleotides are then removed and another different mixture CG* is added to the second nucleotide addition reaction, and the number of incorporated bases “M = 1” is obtained. In each sequencing run, a set of two-digit strings NM can be transformed into encodings. After the template is sequenced twice with AT*/CG* and GA*/TC*, two sets of encodings are obtained sequentially and the sequence can be also determined.

Considering accuracy can be improved by employing dual-base sequencing strategy in which each base is identified twice, a real-time decoding sequencing technology combining dual-base with SBS was proposed (Pu et al., 2014). This approach relies on adding a mixture of two different bases into the reaction each time. The synthetic strands expose free 3′-OH groups that can be continuously extended until no bases in the mixture can be further incorporated. Although each of such reactions provides only an encoding that contains the information about the possible type of incorporated base, the template can be sequenced twice to provide two sets of encodings, from which the sequence can be decoded. For example, in the first extension reaction, a mixture of AT is added to the primed DNA template with the starting sequence AACTGAAAGC. Two bases are incorporated, but they are uncertain, so the incorporated bases are denoted as two encodings (AT). Next, another mixture of CG is added to the second extension reaction, an encoding (CG) is obtained. In this method, these encodings are represented by four-color codes. Nucleotides A, T, C, and G can form sixteen dual-base combinations (AA, AG, AC, AT, GG, GA, GC, GT, CC, CA, CG, CT, TT, TA, TG, and TC), and these sixteen combinations information can be encoded by a four-color code matrix (Figure 5A). When the template is interrogated in two parallel runs using two sets of dual-base additions AT/CG, and GA/TC, two sets of four-color codes with partially defined base composition in each cycle can be obtained. The sequence can be accurately deduced by aligning these two sets of four-color codes (Figure 5A). However, just like SOLiD platform, once an error occurs during sequencing, it may easily give rise to chain decoding errors, leading to wrong base information being decoded. Therefore, in this method, sequencing errors must be corrected by first converting the base sequence of the reference into four-color codes, and then comparing the four-color codes of the reference with that of the original sequence. In addition, Chen et al. (2017) proposed a dual-base sequencing method based on SBS, called ECC sequencing, which rectified errors by aligning three sets of four-color codes obtained in three parallel runs, equivalent to introducing a reference sequence. Here, it was reported that this method obtained a raw sequencing accuracy of 98.1%, and provided single-end, error-free sequences up to 200 bp through an error-correction algorithm. Therefore, this technique has demonstrated its advantages in terms of sequencing accuracy.

However, ECC sequencing technology fails to solve the problem of homopolymer sequencing. Moreover, there is a risk of introducing a longer homopolymer (e.g., in AT/CG dual-mononucleotide flowgram, information for sequence fragments such as TTTTAATTATAAAT, CCGCGCCGGC, etc.), thereby potentially leading to t more errors than traditional single-nucleotide addition (SNA). Therefore, Cheng et al. (Cheng and Xiao, 2022; Cheng et al., 2023) proposed a correctable decoding sequencing technology, in which two kinds of nucleotides, natural nucleotide (denoted as X) and cyclic reversible termination (CRT; denoted as Y*), are added to each reaction cycle. This method is based on the principle that the signal intensities of released identical detection molecules are proportional to the number of incorporated natural nucleotides or/and CRTs. In the first extension reaction, a mixture of two types of nucleotides (X and Y*) is added and the number of incorporated nucleotides “N” is obtained. Then another different mixture (WZ*) is added to the second extension reaction, and the number of incorporated nucleotides “M” is obtained. After two extension reactions, deprotection is conducted and a complete sequencing cycle is completed, before the next cycle then starts. In each reaction cycle, a two-digit code “NM” can be obtained. The decoding algorithm is as follow:

If N = 0, it means that no nucleotide is incorporated. If N = 1, it indicates that one nucleotide is incorporated, but it is uncertain whether it is X or Y, denoted as an encoding (XY). If N ≥ 2, it means that there are (N−1) bases X and an encoding (XY) because the extension reaction is terminated if Y* is incorporated (Wu et al., 2007). If M = 0, it means that the previous extension reaction is terminated by a Y*, so the encoding (XY) in the former reaction must be Y. If M ≥ 1, it can be inferred that the previous extension reaction is not blocked by Y*, so the encoding (XY) in the former reaction must be X (N≠0). Moreover, the sequence information for the second reaction in this cycle indicates that there are (M−1) bases W and an encoding (WZ). In this method, a four-color code matrix is applied to encode base information (Figure 5B). A two-color code denoted an encoding, whereas a single-color code represented an explicit base. For a DNA template with the starting sequence AACTGAAAGC (Figure 5B), a mixture of AT* is added to the first reaction and another dual-base CG* is mixed in the second reaction. N = 2, M = 1 is obtained by the first cycle. It means that two bases are incorporated in the first reaction and one base is incorporated in the second reaction. It can then infer that the first two bases must be AA, because the 3′-end of the synthesized strand that is not terminated can be extended continuously. In addition, M = 1 means that the third base is an encoding (CG). When the template is interrogated in two parallel runs using two sets of dual-base additions AT*/CG*, and GA*/TC*, two sets of four-color codes can be obtained, from which the sequence can be accurately deduced. This strategy can fully resolve issues related to homopolymers, and has great potential in NGS in terms of sequencing decoding, reassembly, error correction and accuracy. Furthermore, it has a theoretical background error rate of less than one artifactual error per 10^5^ nucleotides, which is lower than Sanger sequencing. In addition, this method can judge sequencing information without introducing the reference sequence. Hence, it can realize the effective confirmation of low-abundance sequences.

The read length of dual-base sequencing based on SBS is nearly three times longer than that of single-base addition (Pu and Xiao, 2017). Therefore, dual-base sequencing has high accuracy and can significantly increase the potential read length.

## 5 Improvements in data processing

NGS platforms have been known to sequence hundreds of thousands to millions of DNA molecules in parallel at a time, rapidly generating very large datasets for genomics, epigenomics, and transcriptomic studies. Accordingly, data analysis is quite complex. Until now, many researchers have developed numerous effective algorithms to improve sequencing accuracy.

For example, there are algorithms aimed at improving the accuracy of base calling, which mainly seek to calibrate the dephasing. Since current NGS platforms are based on clonal amplification and sequencing, many identical templates are sequenced simultaneously in a single experiment, encompassing higher requirements for the synchronous extension of DNA molecules. Some templates may not be not extended, while others may have several nucleotides added. This phenomenon is called dephasing, which means that in a clone of the same DNA molecules lose synchronization in the extension reaction. Dephasing has two major components: Lead and lag. Lead means that the reaction occurs in advance, mainly due to contaminating bases in the reaction, while lag means that the reaction is delayed, which is mainly due to insufficient reaction time leading to incomplete extension. Therefore, dephasing is considered to be one of the major problems of read errors in sequencing results. In order to address this problem, researchers have developed dephasing algorithms. Specifically, Erlich et al. (2008) developed a base caller, called Alta-Cyclic, that used machine learning to compensate for noise factors, which was shown to substantially improve the number of accurate reads for sequencing runs up to 78 bases and reduce systematic biases, facilitating confident identification of sequence variants. Chen et al. (2017) introduced the error-correction code (ECC) concept into SBS reactions and corrected the errors through Bayesian probability calculations. They reported that ECC correction can eliminate all errors in the first 200 nt, effectively reducing the cumulative error rate of 250 nt, from 0.96% to 0.33%. Zhou et al. (2020) developed an ordinary differential equation-based model to simulate clonal reactions so as to identify the major factor causing the dephasing, attaining a low error rate in the case of an average read length of 1,000 bp with the dephasing algorithm.

There is also a series of error correction algorithms for the sequence assembly. NGS reads contain far more errors than data from traditional Sanger sequencing, and downstream genomic analysis results can be improved by correcting the errors during the assembly process. The error correction methods can be divided into four basic categories: *k*-mer counting method, probabilistic consistency method, multiple sequence alignment method, and hybrid assembly.

The *k*-mer counting-based error correction methods work by extracting the set of all *k*-mers from the reads, which is termed the *k*-spectrum (Kelley et al., 2010; Yang et al., 2010; Medvedev et al., 2011). The *k*-mers with small Hamming distances among them are likely to belong to the same genomic position. By identifying such a *k*-mer set, alignment is directly achieved without resorting to multiple sequence alignment, and error correction can then be applied by converting each constituent *k*-mer to the consensus (Yang et al., 2013). Typical *k*-mer counting-based error correction algorithms includes: Quack (Kelley et al., 2010), Reptile (Yang et al., 2010), ALL-PATHS-LG (Butler et al., 2008; Maccallum et al., 2009; Gnerre et al., 2011), SOAPdenovo (Li et al., 2010), and EDAR (Zhao et al., 2010).

The idea behind probabilistic consistency-based error correction methods is to determine a threshold and correct *k*-mers whose multiplicities fall below the threshold (Liao et al., 2019). In these methods, choosing the right threshold is crucial because a low threshold can cause too many uncorrected errors, while a high threshold can cause loss of correct *k*-mers. There are a number of probabilistic consistency-based algorithms, such as BayesHammer (Nikolenko et al., 2013), ECHO (Kao et al., 2011), Hammer (Medvedev et al., 2011), and ProbCons (Do et al., 2005), that can effectively correct errors under the condition of uneven sequencing.

The idea behind multiple sequence alignment (MSA) based error correction methods is using sequence alignment to detect and correct erroneous reads by aligning them with each other (Salmela and Schroder, 2011). Reads that share *k*-mers are likely to be similar, while those with high-frequency *k*-mers are likely to be correct and can be used to correct reads with low-frequency *k*-mers. There are many MSA-based algorithms for NGS short reads, such as CABOG (Miller et al., 2008), BWA (Li and Durbin, 2009), bowtie (Langmead et al., 2009), MUMmer (Kurtz et al., 2004), which can accurately correct substitution, insertion and deletion errors of NGS data.

Hybrid assembly works by combining the complementary attributes of different technologies to detect and correct erroneous reads (DiGuistini et al., 2009; Nagarajan and Pop, 2010). For example, the read from 454 platform is longer and the error rate is higher compared to Illumina platform. These longer reads from 454 platform can be used to detect overlaps during assembly, while shorter reads from Illumina reads can be used to detect and correct erroneous reads (Lin et al., 2012). Early hybrid assembly are based on combining the reads from Sanger sequencing and NGS, such as 454, Illumina (DiGuistini et al., 2009). The continuous development of single molecule sequencing has increased the read length and hybrid assembly such as PBcR are also developed (Koren et al., 2012). PBcR corrects erroneous long reads from PacBio using short, high-fidelity reads generated by NGS, such as 454 or Illumina, and then assembles the genome sequence with corrected long reads. The results showed that when using PBcR for hybrid assembly of corn transcriptome, the corrected RNA-seq had very low error rates, with only 0.06% insertion and 0.02% deletion rates. Jason et al. (Miller et al., 2017) also developed a hybrid assembly pipeline called Alpaca, and demonstrated that it is a useful tool for investigating structural and copy number variation within *de novo* assemblies of sampled populations.

In addition, there are numerous algorithms aimed at improving the accuracy of variation detection (Campbell et al., 2008; Quail et al., 2008; Shen et al., 2010; Zagordi et al., 2010; Flaherty et al., 2012; Gerstung et al., 2012). Campbell et al. (2008) developed an algorithm that can differentiate genuine haplotypes of somatic hypermutations from sequencing errors. They demonstrated that this algorithm can detect multiple rare subclones with frequencies as low as 1 in 5,000 copies. Shen et al. (2010) developed a computational tool that detected and accounted for systematic sequencing errors caused by context-related variables in a logistic regression model learned from training datasets. The posterior error probability for each replacement was then estimated by a Bayesian formula that combines prior knowledge of the overall ranking error probability and SNP estimated probability with the results of a logistic regression model for a given replacement. Estimated posterior SNP probabilities can be used to distinguish true SNPs from sequencing errors. They reported a false-positive rate of lower than 10%, with a ∼5% or lower false-negative rate. Gerstung et al. (2012) developed a customized statistical algorithm, called deepSNV, for detecting and quantifying subclonal single-nucleotide variants (SNV) in mixed populations, showing that it can detect variants with frequencies as low as 1/10,000 alleles.

## 6 Limitations and future development of NGS platforms

Currently the prevailing NGS platforms are SBS-based methods that utilize DNA polymerase to extend a new DNA strand and deduce the template sequence by detecting incorporated nucleotides during strand synthesis. Particularly, Illumina serves as the current mainstream sequencing platform (Fuller et al., 2009; Mardis, 2013). However, in terms of SBS technologies, the read length is related to the reaction steps and the type of nucleotides delivered. Excessive reaction steps, or the use of modified nucleotides as substrates, can result in significantly reduced synthesis efficiency, thus affecting the read length. Meanwhile, as the sequencing reaction progresses, the possibility of dephasing becomes higher and higher, leading to a sharp increase in fluorescence noise, which in turn leads to the premature termination of the sequencing reaction. The final sequencing accuracy has shown to range between 99.2% and 99.74% for Illumina/Solexa, 99% for Roche/454, and 98.22% for Ion Torrent. However, some highly sensitive genetic analyses have demonstrated that the true mutation frequency in normal cells may be much lower, with estimates of pre-nucleotide mutation frequencies being generally between 10^−8^ and 10^−11^ (Cervantes et al., 2002; Roach et al., 2010). Therefore, most mutations seen in normal human genomic DNA by NGS platforms are still likely to be technical artifacts. Moreover, due to high error rate of NGS platforms, the identification of somatic variants that are present in a single copy, or a few copies (if clonally amplified), poses many problems. Although NGS technologies have been widely used in biology and medicine, there is much room for improvement in terms of sequencing accuracy and read length.

**FIGURE 6:**
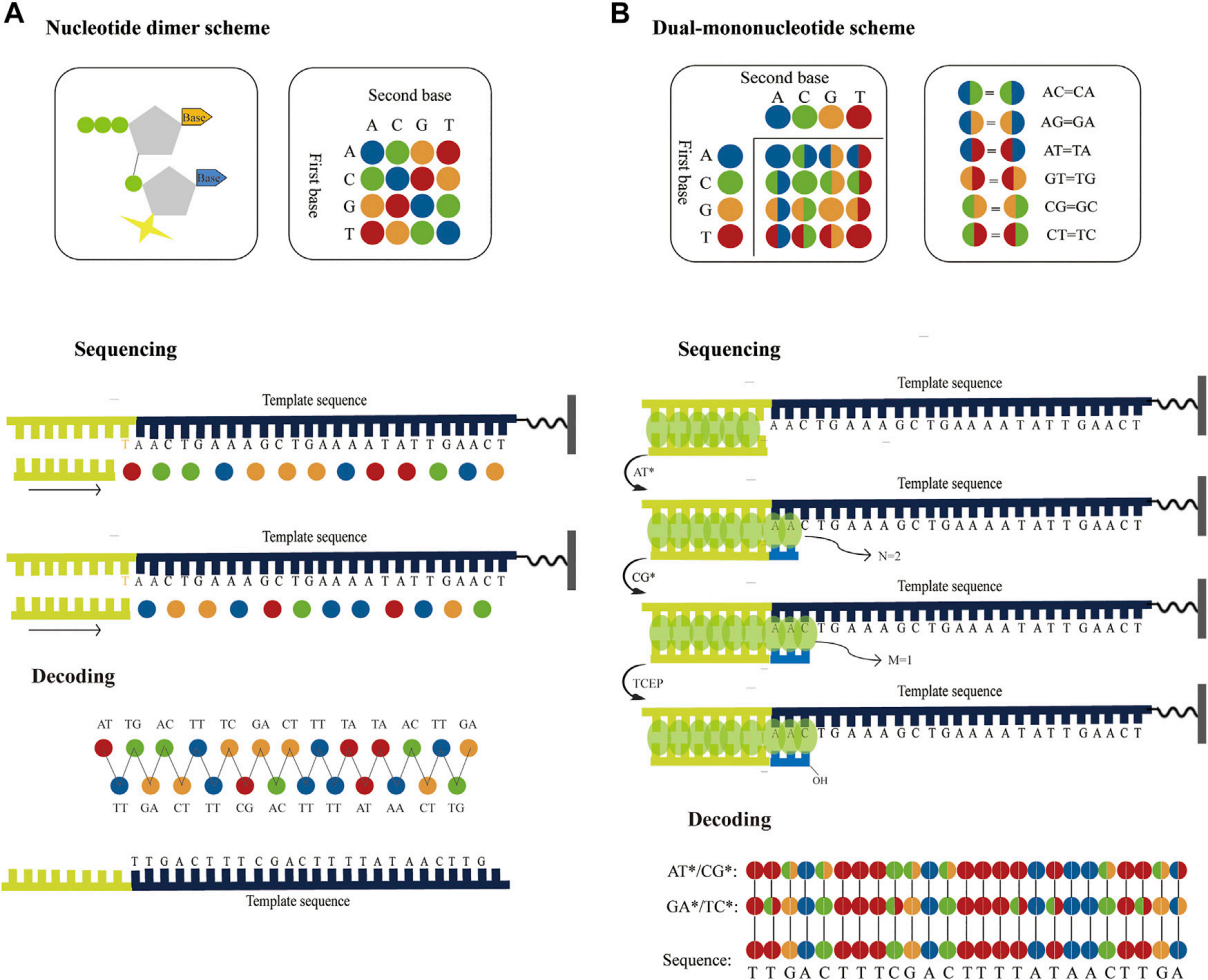
Possible strategies to improve the accuracy of the surface fluorescence sequencing platforms based on chip amplification. **(A)** Application of dual-base sequencing on the surface fluorescence sequencing platforms based on chip amplification. The four different fluorophores represent 16 nucleotide dimers, respectively. The nucleotide dimer complementary to the template is incorporated using DNA polymerase and is imaged to identify these two bases. Then the fluorophore is cleaved off and another round of extension begins. This cycle is repeated several times until the complete template is sequenced. Another round of sequencing occurs with a primer that has one base longer than the previous primer. Therefore, two sets of four-color codes can be obtained from two parallel runs, from which the sequence information can be then decoded. **(B)** Application of the correctable decoding sequencing on the surface fluorescence sequencing platforms based on chip amplification. The number of incorporated nucleotides in each extension reaction is determined by quantitative staining double-stranded DNA with fluorescent dyes. In the first extension reaction, a mixture of AT* is added to the primed DNA template. DNA polymerase incorporates two bases to pair the first two bases and generates two fluorescent intensities. Next, another mixture of CG* is added to the second extension reaction, one base is paired with the next one base to generate one fluorescent intensity. The template sequence information can be deduced through two parallel runs using different dual-base addition.

In principle, using nucleotide dimers as substrates for SBS (Joshi et al., 2012; Damha et al., 2015) could theoretically achieve similar sequencing accuracy for SOLiD platform. Therefore, if dual-base sequencing based on SBS can be applied to surface fluorescence sequencing platforms based on chip amplification, such as Illumina platform, both long read length and high accuracy can be achieved. For example, a nucleotide dimer with a fluorophore at the 3′ ends can be designed and synthesized, of which 16 possible nucleotide dimers correspond to four different fluorophores, where each fluorophore represents a subset of the four nucleotide dimers. During sequencing, a nucleotide dimer complementary to the template is incorporated and the slide is imaged to identify these two bases. Unbonded nucleotide dimers are then removed and the fluorophore is cleaved off thereby preparing for another round of extension. This cycle is repeated several times until the complete template is sequenced. Another round of sequencing occurs with a primer that has one base longer than the previous primer. Therefore, the template sequence information can be deduced through two parallel runs using ladder primer sets (Figure 6A). Similar to SOLiD platform, this method initially converts the base sequence of the reference into four-color codes, and then compares the four-color codes of reference with that of the original sequence to correct sequencing errors.

The correctable decoding sequencing strategy is, in principle, also compatible with surface fluorescence sequencing platforms based on chip amplification. For example, if the fluorescence intensity of the extension region is proportional to the number of synthetic nucleotides, and when a single base can be distinguished, the specific number of incorporated nucleotides in each extension reaction can be directly determined. Thus, the specific base or encoding information of the extension reaction can be inferred (Figure 6B). After the template is sequenced twice with two rounds of dual-base addition, two sets of base-encoding strings are obtained sequentially, and the sequence can then be accurately deduced. Since the two rounds of sequencing information have an inherent correction function between each other, this method can greatly improve sequencing accuracy. Therefore, this method may bring about the discovery of low-abundance mutations in sequences for scientific research and clinical practice, which holds potential and being the most accurate high-throughput DNA sequencing approach. In theory, the aforementioned sequencing techniques can be attained as long as suitable enzymes or small fluorescent molecules are available. Although no relevant reports currently exist, as research in this area continues to develop, researchers will be able to better understand and further improve upon the technology in the future. As a result, the direction or potential for future high-throughput DNA sequencing research may be identified.

## 7 Conclusion

NGS technologies have achieved remarkable progress as affordable and fast sequencing platforms, which are currently mainstream sequencing platforms, and have been widely used. Although many aspects of sequencing have been improved, compared with Sanger sequencing, low accuracy and short sequencing read length continues to be problems. NGS platforms cannot generally be used to detect rare variants because of the associated high error rate. In addition, existing NGS platforms are unable to judge the information of a single read, thereby limiting their clinical application in the determination of low-abundant mutations. Fortunately, a dual-base sequencing strategy may provide help achieve high accuracy sequencing. Further research may uncover novel sequencing solutions to continue to expand the scope of sequencing application.
